# Barriers to reporting clinical errors in operating theatres and intensive care units of a university hospital: a qualitative study

**DOI:** 10.1186/s12912-021-00717-w

**Published:** 2021-10-27

**Authors:** Sedighe Ghobadian, Mansour Zahiri, Behnaz Dindamal, Hossein Dargahi, Farzad Faraji-Khiavi

**Affiliations:** 1 School of Health, Ahvaz Jundishapur University of Medical Sciences, Ahvaz, Iran; 2 Department of Health Services Management, Ahvaz Jundishapur University of Medical Sciences, Ahvaz, Iran; 3 Health Information Management Research Center, Tehran University of Medical Sciences, Tehran, Iran; 4 Social Determinants of Health Research Center, Ahvaz Jundishapur University of Medical Sciences, Ahvaz, Iran

**Keywords:** Hospital errors, Error reporting impediments, Expressing errors

## Abstract

**Background:**

Clinical errors are one of the challenges of health care in different countries, and obtaining accurate statistics regarding clinical errors in most countries is a difficult process which varies from one study to another. The current study was conducted to identify barriers to reporting clinical errors in the operating theatre and the intensive care unit of a university hospital.

**Methods:**

This qualitative study was conducted in the operating theatre and intensive care unit of a university hospital. Data collection was conducted through semi-structured interviews with health care staff, senior doctors, and surgical assistants. Data analysis was carried out through listening to the recorded interviews and developing transcripts of the interviews. Meaning units were identified and codified based on the type of discussion. Then, codes which had a common concept were grouped under one category. Finally, the codes and designated categories were analysed, discussed and confirmed by a panel of four experts of qualitative content analysis, and the main existing problems were identified and derived.

**Results:**

Barriers to reporting clinical errors were extracted in two themes: individual problems and organizational problems. Individual problems included 4 categories and 12 codes and organizational problems included 6 categories and 17 codes. The results showed that in the majority of cases, nurses expressed their desire to change the current prevailing attitudes in the workplace while doctors expected the officials to implement reform policies regarding clinical errors in university hospitals.

**Conclusion:**

In order to alleviate the barriers to reporting clinical errors, both individual and organizational problems should be addressed and resolved. At an individual level, training nursing and medical teams on error recognition is recommended. In order to solve organizational problems, on the other hand, the process of reporting clinical errors should be improved as far as the nursing team is concerned, but when it comes to the medical team, addressing legal loopholes should be given full consideration.

## Background

Hospital errors are among the key challenges of the health care service industry worldwide. In the USA, for instance, nearly one hundred thousand people lose their lives each year as a result of medical errors, the eighth leading cause of death among patients in this country [1–3]. Hospital errors also impose huge financial burdens on health care systems annually [4]. According to one study, the National Health Service (NHS) spends five hundred million pounds annually for the additional days patients spend in hospital due medical errors [5].

However, obtaining accurate statistics related to hospital errors is difficult in most countries and varies from one study to another. It is believed that only through the recognition of the rights of patients and an analysis of cases where these rights have been violated can such errors be identified [6]. Obtaining an overview of medical errors is more difficult in developing countries. This is not because there are no medical errors in these countries. Rather, it could be attributed to the improper reporting of these errors, the lack of an adequate records system, and paucity of research in this area [7].

According to a study carried out in Tehran hospitals, the average number of medical error committed by each nurse during a three-month period was 19.5, but only one-third of these errors were reported [8]. The real frequency of hospital errors is not available in Iran, but experts speculate that given the large number of medical error lawsuits referred to the medical council, the medical teams at hospitals might have a higher rate of errors [9]. In addition, every year, huge sums of money have been claimed to be spent by the Iranian Ministry of Health and Medical Education in Iran for the medical care of patients hospitalized because of medical errors, a claim whose accuracy might be revealed in future [10].

The World Health Organization considers reporting and disclosing errors as a useful learning strategy and the basis for the development of strategies to prevent future errors [11]. Error reporting has a positive effect on patient safety and is a stimulus for change in the process of care. It contributes to the improvement of culture, knowledge, and attitudes towards voluntary anonymous incident reporting [12, 13]. In addition, according to previous studies, reporting and disclosing errors is the first step towards identification of the reforms needed in this respect [14]. However, what occurs in reality is that members of medical teams often fail to report workplace errors [15]. According to one study, lack of reports related to medical errors raises concerns about the number of errors actually occurring in this context, which highlights the need to study and identify the barriers to reporting medical errors [6]. Another study has introduced disclosure of hospital errors as the main cause for the reduction of these errors in health care organizations [9].

According to the studies that have been carried out on this topic, the most important barriers to reporting clinical errors are as follows: regarding the person committing the error as incompetent by other members of the medical team, fear of compromising one’ s position [16], lack of legal and financial support for medical personnel who commit errors [2], the time-consuming process of reporting an error [17], reluctance of members of the healthcare team to report errors [9], lack of an understanding of the severity of the error [6], chastisement by directors and supervisors [18], and the recording of the error in the employee’s personnel file [16]. In study of Heard et al., worry about disciplinary action, colleagues’ blame, unsupportive colleagues, and unwillingness to discuss errors in hospital meetings have also been identified as major obstacles in reporting clinical errors among medical groups [19].

The occurrence of clinical errors varies across different departments of a hospital. Statistics show that 50% of all adverse hospital errors occur in operating theatres. It has also been reported that up to 10% of the mortality rates are due to postoperative deaths, accounting for about one million deaths worldwide [20, 21]. In terms of the frequency of medical errors, operating theatres are followed by intensive care units, which account for 15 to 21% of the maximum harm done to hospitalized patients. Nevertheless, the average harm to patients hospitalized in wards is from 10 to 16% [22]. In addition, according to previous studies, the mortality rate of patients in intensive care units as a result of hospital errors is 41% higher than that of all other patients hospitalized in different wards [23].

Operating theatres and intensive care units of university hospitals are characterized with the high concentration of services delivered to patients. They also involve patients hospitalized with specific conditions and a variety of treatment groups. These reasons make these two parts of the hospital more prone to higher risks and make the incidence of medical errors more likely. Therefore, controlling medical errors needs to be set as priority in managerial and nursing action plans [9]. Within medical error control process, identifying and minimizing impediments of reporting clinical errors relying on lessons learned by others play a crucial role in decreasing the odds of clinical errors [24]. Therefore, the current study aimed at identifying the barriers to reporting clinical errors in the operating theatre and the intensive care unit of a university hospital in Ahvaz, southwest of Iran.

## Methods

### Study design

This was a qualitative research using content analysis of semi-structured interviews. The purpose of content analysis is to organize and elicit meaning from the collected data and to draw realistic conclusions from it [25]. In addition, for data triangulation and increasing the credibility and validity of the findings obtained from the content analysis, a member of the hospital quality improvement committee was invited to focus group sessions. Through this joint sessions, the hospital authorities found out about existing barriers to reporting clinical errors.

### Participants and sampling

The population of the present study included all the staff (nurses and doctors) working in the operating theatres and intensive care units of a university hospital, Ahvaz, Iran. Participants were eligible according to inclusion criteria provided that their career was concerned howsoever with clinical errors and they were willing to participate in the study. The study participants were selected from among the staff of a general surgery operating theatre, three specialist operating theatres (including cardiology, angiography and organ transplant), a general intensive care unit, and three specialized intensive care units (including cardiology, general surgery and neurosurgery departments). Participants’ career experience ranged from 5 years to 26 years. Specialists and postgraduate medical students (residents and fellows) in the general surgery, anaesthesia, neurosurgery, and vascular surgery voluntarily contributed to the study. Thirty nurses and 15 physicians were selected by purposive sampling method. The purposive sampling technique, also known as judgment sampling, is the deliberate choice of an informant due to the qualities the informant possesses. In this context, the researcher decides what needs to be known and sets out to find people who can and are willing to provide the information by virtue of knowledge or experience [26]. In the next steps, some of the informants who have more experience will be asked to participate in focus group sessions to confirm the findings. Regarding education and experience of this research participants, informants were a part of the focus groups from the beginning. In our study, this focus group consisted of 12 members including a nursing education supervisor, a quality improvement nurse, representative/head nurses of the studied wards, representatives of surgery and anaesthesia residents, and experienced surgeons.

### Data collection

Data collection was carried out via semi-structured interviews. Two researchers with good experience in this field were selected as interviewers. They started the interviews after setting an appointment with individuals who met the inclusion criteria. The interviews were conducted face to face. Interviewees were asked to answer two key questions: “What prevents you or your colleagues from reporting clinical errors?” and “What factors make you or your co-workers more motivated to report clinical errors?” The interviews lasted from 30 to 45 min and continued until data saturation. This procedure took 3 months. With the consent of the participants, all interviews were recorded using a tape recorder, were carefully listened to, and then transcribed verbatim. An attempt was made to conduct the interviews without bias and to write only the whole content.

### Data analysis

The data were analysed based on Colaizzi’s method [27]. Data analysis was recorded by listening to the interviews and producing transcripts. In the next stage, meaning units were identified and codified. Codes with the same meaning were then grouped under one category, and from these categories, the themes (i.e., the main existing problems) emerged. Finally, a panel consisting of four experts in qualitative content analysis reviewed and analysed the codes, organized the categories and verified the themes. Data coding was done at two different occasions by two different coders, and then the codes were compared and discrepancies were resolved.

### Scientific trustworthiness of the results

We checked the trustworthiness of the data according to Lincoln and Guba’s four-criteria, namely credibility, dependability, confirmability, and transferability [28]. Furthermore, to evaluate and enhance the credibility of the findings, sampling continued until data saturation. Transferability of data was provided by offering a comprehensive description of the topic, the participants, data gathering, and data analysis. Then, to increase the dependability of the research results, we used external checking. The confirmability of the findings was increased via investigator triangulation [29].

Finally, after categorizing the collected data and checking them with participants, they were presented in focus group sessions for final confirmation. Focus group discussion is a technique where a researcher assembles a group of individuals to discuss a specific topic, aiming to draw from the complex personal experiences, beliefs, perceptions and attitudes of the participants through a moderated interaction. This is perceived to be a “cost-effective” and “promising alternative” in qualitative studies [30].

In order to create consistency, we invited individuals with various expertise in nursing and medicine. In our study, we merged our focus group sessions with those of the hospital quality improvement committee, through assistance from the head of hospital. The study involved four focus group sessions held every other week, each lasting about 120 min. The meetings were directed by 2 researchers. To end with, the focus group in this study served three aims: first, checking the accuracy of understanding, second, sharing the first analysis with the expert informants to gain credibility, and third, informing the quality control and having their applicable points.

### Ethical considerations

After receiving approval from the Ethics Committee of Ahvaz Jundishapur University of Medical Sciences (IR.AJUMS.REC.1396.182), coordinating with the participants’ supervisors, and receiving informed consent, the researchers began conducting the interviews. The participants were clearly briefed that they had the right to withdraw from the study at any time even after the informed consent had been signed. Each interview was conducted individually in an appropriate office that was made ready for these interviews. The participants were also briefed about the aims of the study and confidentiality of their personal information. In addition, prior to the interview sessions, informed consent was taken from the participants for recording the interviews. In order to keep the interviewees anonymous and to distinguish them from one another in presenting the findings of the study, a code was assigned to each one of them. We followed the ethical protocols in accordance with the relevant guidelines and regulations.

## Results

Twenty-nine codes in 10 categories were extracted from the interviews. These categories were classified in 2 themes, namely individual problems and organizational problems. These themes are discussed from the perspective of the two groups participating in our study, namely the nurses and the physicians. Individual problems from the nurses’ perspective included 7 codes and 2 categories while organizational problems from their perspective consisted of 9 codes and 4 categories. From the physicians’ perspective, individual problems contained 5 codes and 2 categories whereas organizational problems included 8 codes and 2 categories. Two categories, namely educational and attitudinal problems, were extracted from interviews with both nurses and doctors. However, these two categories were deducted from different codes, namely remarks and implications. Therefore, we did not merge them to prevent losing some valuable remarks and perspectives.

Table 1 shows nurses’ views on individual problems.

**Table 1:** Individual problems inhibiting the reporting of clinical errors by the healthcare team from the perspective of nurses

Theme	Categories	Codes	Semantic units
Individual problems	Problems associated with the individuals’ training	Lack of knowledge about types of error	1. Being unaware of what counts as an error. 2. The diversity of clinical errors
		Failure to provide continuous and in depth training of staff	1. Accepting numerous novice medical trainees in the education system, and the need for on-going education 2. Forgetting some of clinical procedures used less frequently may result in unawareness of a clinical error 3. Failure to use different and modern training methods in order to motivate staff to actively participate in training programs
		Failure to complete all courses at university	1. Lack of sufficient scientific information related to the use of potentially hazardous drugs or required dosages and inability to identify the occurrence of the error. 2. Being unaware about the benefits of reporting clinical errors
		Flaws in hospital training	1.All members of the nursing staff are not equally present during training sessions 2. Staff are not aware of the effects of clinical reporting and follow-up on the enhancing of patient well- being and the enhancing of the quality of care service provided
	Problems associated with the individuals’ attitude	Natural inclination to cover-up an error	1. It is in the interest of most people to maintain the appearance and image of their occupation
		Complications resulting from reporting errors	1. Upon observing any drug interactions, the staff member must check the drug manufacturer’s leaflet to identify which ingredient of the drug causes the reaction which is in most cases beyond the competence of the medical team. 2. Residents and interns believe that the reporting of clinical errors is the sole responsibility of the ward officials 3. A clinical error might be repeated several times by the staff yet since no reporting is done, the clinical error becomes routine and looks trivial.
		Fear of jeopardizing job security	1. Sometimes employee’s occupational errors or oversight are recorded as infraction in his or her job history, which may have repercussions for the person in the future.

Many of the nurses considered educational problems as the most important obstacle to reporting errors. “I do not know exactly what errors need to be reported and followed up” (Participant 5). “At university, we were not taught well on patient safety issues” (Participant 13). “I have just started my job in the hospital, and I am not aware of the error reporting process” (Participant 24). Some nurses also reported attitudinal problems. “Reporting an error is very time consuming and I do not have enough time to do it” (Participant 8). “No one reports any error because they are afraid of being seen as a culprit” (Participant 19).

Table 2 lists the categories and codes related to the theme of organizational problems from the perspective of nurses.

**Table 2:** Organizational problems inhibiting the reporting of clinical errors by the healthcare team from the perspective of nurses

Theme	Categories	Codes	Semantic units
Organizational problems	Motivational problems related to reporting clinical errors in a system	Failure to obtain expected results	1. Staff members do not obtain the expected outcomes from reporting clinical errors. 2. Inspiring people who have no direct connection with the recording and reporting of clinical errors. 3. Failure to take corrective measures taken after reporting clinical errors 4. Failure to provide financial incentives for reporting clinical errors 5. Concessions should be proportional to the type of clinical error reported
		Suspicion of colleagues and all members of the healthcare team	1. The individual committing an error is ostracized by colleagues 2. The individual committing an error feels that other colleagues attribute the clinical error to his/her negligence
		Censure by nurse supervisor	1. The individual committing an error is severely criticized by ward supervisor. 2. Not being cooperative with the individual committing an error (e.g., rejecting leave requests) 3. Though the error might be minimal, it will have long-term consequences and repercussions.
		Fear of physicians’ managerial role or positions	1. Clinical errors committed by physicians affiliated with university or members of the board of directors of the hospital are not reported. 2. Failure to report a clinical error due to a physician’s negligence 3. Fear of censure by the attending physician once the clinical error is reported.
	The procedural problems of the system	Workload of the medical staff	1. Lack of sufficient time to record and report errors due to the shortage of ward staff 2. An increase in patients seeking treatment at university hospitals especially after the Health Reform Act and the unproportionate ratio of patients to health care staff even after more than three rounds of nursing and medical care staff employment. 3. Failure to have a skilled staff member in patient
		Problems with reporting clinical errors	1. Current forms for the recording of clinical errors are extensive and complicated 2. The majority of the medical staff are unaware of the procedure for reporting clinical errors 3. Unavailability of clinical error report forms during all hospital shifts. 4. The faulty and lengthy process of recording the clinical error through clinical error databases
	Structural problems	Absence of an HSE manager at the hospital	1. An HSE officer in addition to a supervisor should be assigned to each shift.
		Absence of a psychologist in high-risk wards	1. Failure to hire a trained psychologist in high-risk wards who is responsible to predict possible clinical errors in the ward and to mentally prepare the staff for dealing with potential clinical errors.
	Managerial problems	Indifference of top management towards dealing with clinical errors	1. Failure to control the procedure and method of reporting of clinical errors in the ward by ward supervisors and directors of shifts. 2. Failure to create an HSE culture 3. The shortage in ward staff brings on heavy workload in university hospitals, making administrative staff set lower expectations from the health care staff to focus on the patients well-being in lieu of reporting and following up clinical errors. By the same token, the personnel fail to pay due attention to the aforementioned issues. 4. Lack of transparency in the use of recorded clinical errors (especially when used for the improving of quality of service) 5. Administrative staff may exert subjective discretion when a clinical error occurs.

Some nurses mentioned motivational problems to contribute to the failure to report errors. “If they report an error, no one encourages them and the head nurse may even become hostile to them” (Participant 14). Some nurses considered procedural problems to be very important. “We are facing a shortage of nursing staff in the hospital, which leads to negligence in the workplace and failure to identify or even report clinical errors” (Participant 26). A number of nurses reported structural problems. “There is no one in the hospital who can act as a patient safety manager and guide us” (Participant 11). Management problems were also among the problems mentioned by nurses. “The issue of reporting clinical errors is not important to some nurses because top management officials do not take any specific action to control these errors” (Participant 6).

Table 3 shows the barriers to reporting clinical errors (individual problems) from physicians’ perspective.

**Table 3:** Individual problems inhibiting the reporting of clinical errors by the healthcare team from the physicians’ perspective

Theme	Categories	Codes	Semantic units
Individual problems	Educational problems	Lack of emphasis on ethics	1. Poor training on how to identify clinical errors and the type of action required upon the occurrence of such errors 2. Not accepting some clinical errors as uncontrollable events 3. Failure to adopt solutions suggested in dependable medical textbooks such as the holding regular self-expression meetings where one’s clinical errors are discussed and using the experience of others
		Preference of a practical to a theoretical approach in conducting research	1. Due to the high workload, trainees have less time to study, and most of the academic training done is carried out during bedside visits, thus little research activity occurs in the process
	Attitudinal problems	Unwillingness to disclose errors	1. Even in cases where the lack of equipment or failure of a device causes clinical errors, physicians fail to report them.
		Reporting clinical errors is within the nurse’s duties	1. Most physicians consider recording and reporting clinical errors as being related solely to a nurse’s duties.
		Financial interests	1. Physicians fear the risk of losing their clientele after reporting clinical errors.

Some physicians cited two codes under educational problems associated with lack of reporting errors, namely Lack of emphasis on ethics and Preference of a practical to theoretical approaches in doing research. “Some nurses are not able to communicate with the patient and his family, which leads to the refusal to disclose the error” (Participant 4). “A comprehensive definition of clinical errors has not been provided to the nursing staff” (Participant 10). Some physicians also expressed attitudinal problems. “Physicians have to cover up their mistakes to maintain their reputation if they make a mistake” (Participant 12). “Recording and reporting clinical errors is within the scope of nurses’ work and does not concern us [physicians]” (Participant 7). Financial interests were also important to some physicians. “If doctors expose clinical errors, they may lose their patients” (Participant 1).

Table 4 shows the barriers to reporting clinical errors (organizational problems) from the perspective of physicians.

**Table 4:** Organizational problems inhibiting the reporting of clinical errors by the healthcare team from the physicians’ perspective

Theme	Categories	Codes	Semantic units
Organizational problems	Problems in the hospital	Severe chastisement by senior physicians	1. The tendency to severely chastise the interns has resulted in failure to report clinical errors unless catastrophic ones. 2. In most cases, senior residents on the ward fear being chastised by their professors and therefore treat the interns with undue harshness.
		Unconventional punishment	1. Even a minor clinical error will have severe repercussions and will bring on the distrust of professors and other physicians.
		Lack of close monitoring in high risk cases	1. In case of an emergency or during the night shift, professors rely solely on reports provided by aides.
		Long shifts and high workloads	1. Trainees may sometimes work in consecutive shifts over several days causing fatigue which minimizes their ability to report clinical errors.
		Lack of standard forms for reporting clinical errors	1. To date, there have been no already prepared forms for recording and reporting clinical errors.
	Problems related to external factors	Legal loopholes	1. Failure to obtain informed consent from patients for therapeutic intervention and its associated risks at training hospitals. 2. The majority of patients visiting university hospitals are often financially disadvantaged.
		Lack of compatibility between legal sentences and standards	1. Lack of the needed devices and facilities for performing medical procedures at university hospitals or the faulty operation of the existing devices may result in a clinical error during an essential medical procedure, but the law imposes penalties similar to those in developed countries with standard equipment and devices.
		Subjective view	1. The physician is considered by law as the sole perpetrator and condemned as such.

Some of the identified problems were at the hospital level and some at levels higher than hospital. Problems raised by physicians at the hospital level were: “Due to the severe reprimand of surgical assistants by professors, we report clinical errors only in very catastrophic cases and do not usually report minor errors” (Participant 2). “There is no already prepared forms for recording and reporting clinical errors, which will lead to disregarding error reporting” (Participant 7). “We receive irrational penalties from professors for very minor errors” (Participant 14). Some physicians pointed to problems at levels higher than the hospital. “The educational system has been established with the aim of training students thus an intern is likely to commit errors during such a period; however, no error committed in a university hospital is accepted by law” (Participant 8).

**Fig. 1:**
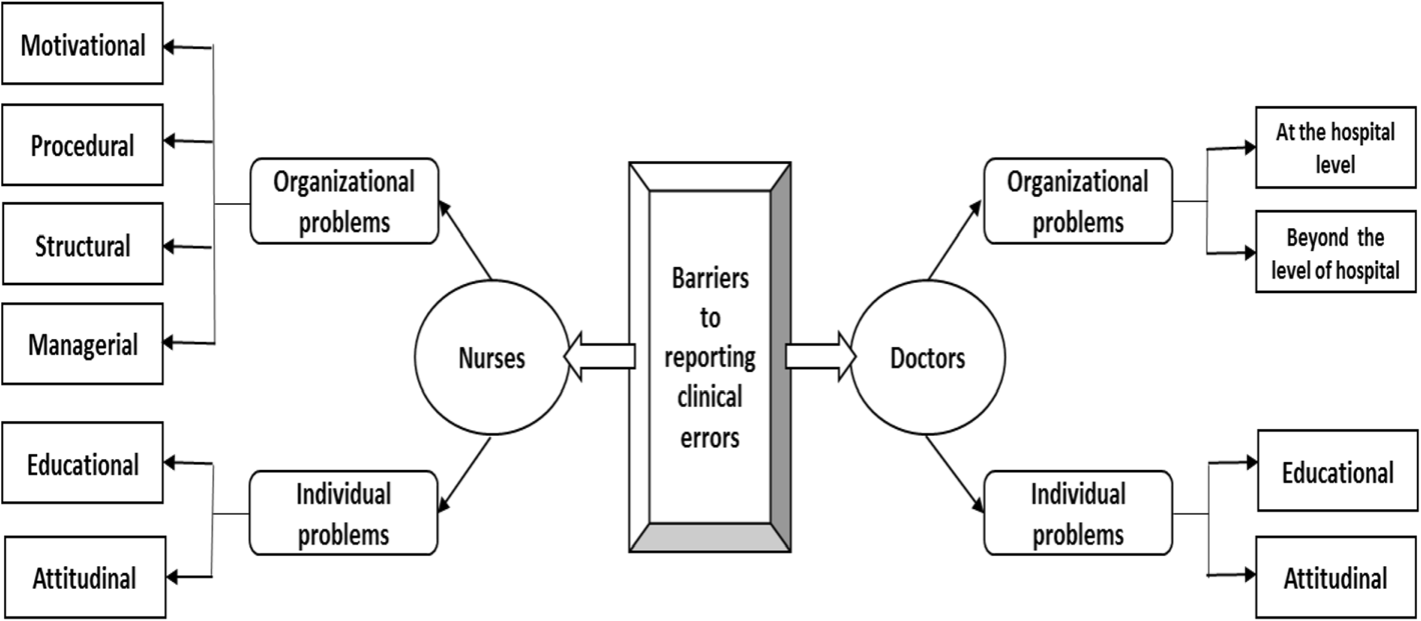
Impediments to reporting errors from the perspective of Nurses and Physicians

Figure 1 shows the overall results of the factors limiting the reporting of hospital errors by Nurses and Physicians.

## Discussion

In the current study the reasons for clinical errors and the manner in which they occur and are identified in a university hospital were elicited through in-depth semi-structured interviews with medical and nursing groups. As far as the nursing staff were concerned, the most significant obstacle impeding the reporting of clinical errors was related to hospital systems which was categorized under organizational problems in our study. On the other hand, physicians identified this problem within external problems related to the country’s health care system and beyond the boundaries of the hospital itself.

In most cases, the interviewed nurses sought to change the prevailing attitudes and practices. However, the physicians expected a modification of the policies related to addressing clinical errors in university hospitals on a governmental level. All interviewees also called for methods for dealing with individual reprimands, continuous training on identifying clinical errors, and methods of self-reporting.

The two main categories of individual problems and organizational problems were specified as the main problems elicited from the interviews with all members of the treatment group. The surgeons and physicians in operating theatres and the nursing staff had the highest rate of individual problems which revolved especially around lack of information about various errors due to the variety of clinical errors and defects in the current training carried out at university hospitals.

According to a study in Iran, 73% of the failure to report clinical errors was due to not being aware of the error itself [31]. Nurses’ unawareness of the definition of hospital errors is mentioned as the main cause for failure to report clinical errors [32]. Also in other studies, one of the main barriers to reporting and disclosing errors was: lack of a unified and standard definition regarding what makes an error a serious one and when errors are eligible for reporting and disclosing [33, 34]. Uncertainty about what exactly constitutes an error also reduces the rate of reporting. This in turn has an impact on whether a hospital employee decides a particular event is an error and whether or not the employee files a report [35]. Also employees do not regard as important those errors committed long ago, and for this reason, do not feel the necessity to report them [36]. Some studies have shown that reports of clinical errors filed by the staff is far fewer than those reported by the same staff after receiving training on how to identify hospital errors [1, 31]. Alijanzadeh et al. and Beiranvand et al. have recommended training of hospital staff in identifying common clinical errors in hospitals [17, 37]. Another study in Iran showed that about half of nurses (49%) had experienced an error/adverse event in the intensive care units in the past year, and most of them did not report it properly (70.9%), despite the fact that about half of them (48.2%) had participated in a reporting errors workshop since they started working in intensive care [38]. Although these studies provide useful evidence, our study sought to shed light on some unclear perspectives of the reasons of not reporting medical errors. These included failure to complete all course hours at university, physicians’ views on the assignment of reporting errors to nurses, failure to obtain the expected results, and absence of a psychologist in high-risk wards. However, what all these studies have in common is the topic of training. In fact, during their undergraduate programs and during continuous training programs in hospitals, the medical staff should receive training on identifying common and life threatening clinical errors and on efficiently reporting them. This will contribute to the development of a culture of self-reporting clinical errors and will ultimately result in a decline of such errors in the hospital.

In the nursing team, the most significant codes categorized under personal perspective included: fear of losing one’s job, concern about the repercussions of reporting errors, the inherent tendency to conceal one’s errors, and the strive to maintain one’s social status, all of which have been discussed in previous studies. Heidari states that 38% of the failure to report clinical errors are due to the person’s fear of losing his/her job [31]. In another study performed in university hospitals in Iran, 46% of the failure to report clinical errors occurred as a result of one’s fear of jeopardizing his/her job [2]. With respect to concerns about the repercussions of reporting errors, one study in Texas where reporting is mandatory found that both physicians (40%) and nurses (30%) were worried about the lack of anonymity in reports and that the reports would be used punitively against the individual who submitted the report [39]. Another study on nurses in South Korea found that 32% of them were worried that their errors would be filed [40]. Nurses have also reported that the disclosing and reporting of practice errors could damage patients’ trust in nurses’ competencies and might lead to litigation [41]. Based on the aforementioned reasons, one may draw the conclusion that in order to minimize the personal perspective of the staff in medical and treatment centres regarding clinical errors, a systematic approach must be instigated in order to increase the reporting of clinical errors in such centres.

As far as organizational problems were concerned, the participants indicated three subcategories, namely the motivational factors for reporting clinical errors, structural problems, and managerial problems. Most of the interviewees’ statements and perceptions focused on organizational problems of reporting errors, and these problems were regarded as the main reasons for the occurrence of clinical errors.

Nurses stated that the absence of any sort of feedback after reporting a clinical error is another reason for the failure to report clinical errors in hospitals. This issue was categorized under motivational problems in reporting clinical errors. In a study carried out in southern Australia by Evans et al., it was seen that the lack of feedback after the disclosure of a clinical error resulted in further reporting of clinical errors [42]. Some nurses believed that positive feedback could be introduced in form of a financial incentive, some mentioned receiving other incentives such as leave bonus, yet others indicated the implementation of change as a sign of suitable feedback for minimizing clinical errors. In a study carried out by Elder et al., it was observed that receiving financial rewards motivates the reporting of clinical errors [14]. Reporting clinical errors in the health system is an effective means of identifying and resolving of clinical errors in the hospital. Nevertheless, it is believed that creating the necessary incentives for nurses to report clinical errors or for those proposing solutions to reduce or eliminate clinical errors may set the stage for creating a workplace in which reporting these errors is based on a systematic approach.

Wagner et al. identified several barriers to reporting and disclosing practice errors. These included negative reactions and feedbacks by head nurse managers, encouragement of a selective reporting of incidents, ignoring nurses’ clinical reasoning and judgment in handling error reports, anonymity and confidentiality issues, concerns over being sued and reprimanded by administrators at the workplace, and endangering nurses’ professional reputation [33]. Nurse managers are responsible for encouraging error disclosure through policy making, creation of a supportive culture, and encouraging nurses to consider ethical values via provision of care, education, and mentorship [43]. It should be noted that by converting negative feedback into positive feedback, it is possible to provide the basis for voluntary error reporting by health care staff.

Another influential factor contributing to the failure in reporting clinical errors was absence of a Health, Safety and Environmental (HSE) manager on hand in the hospital in order to supervise the implementation of the necessary scope of services related to the safety and well-being of the patient and to provide the staff with training programs dedicated to the reporting of clinical errors in addition to following up such reports. These problems, which have not been discussed in most previous studies, were categorized under structural problems during the analysis carried out by the expert panel. In recent years and based on the evaluation standards implemented in hospitals throughout the country, it has been advised that an HSE department be established in hospitals. However, due to staffing problems, the majority of hospitals have not been able to establish such a department. According to Seidi et al.’s study on nurses’ perspectives regarding impediments in the reporting of clinical errors, most of the participants reported that forgetting to report the clinical error was the main reason for not reporting errors. This in itself is related to the lack of an official responsible for the supervising of such reports and recording of the number of reports in hospitals [44].

According to the participants in our study, lack of a standard reporting procedure for reporting clinical errors was another important obstacle hindering adequate reporting of clinical errors. The unclear process of reporting clinical errors is also cited in other studies in Iran [17, 44]. In addition, lack of adequate knowledge of the existing reporting process and the distrust in the current digital systems for reporting have been found to be other barriers to error reporting [33, 34]. Uribe et al. surveyed physicians and nurses at a medical centre located in the Midwest United States about barriers that could be modified in order to facilitate error reporting. The modifiable barriers they identified were the structure and the processes for reporting errors as well as the lack of education about errors [45]. Give the new accreditation system platform which has been in effect by the Iranian Ministry of Health since the last decade, establishing a unified and effective system for reporting medical errors will not be far from reach. By creating a standard system to record and track clinical errors by senior physicians, there will be a reduction in the type of reporting carried out subjectively by other medical professionals and the health care system, which will, in turn, reduce the rate of clinical errors. Moreover, it will aid in the identifying high-risk areas in which most surgical assistants are likely to commit a clinical error and thus create the necessary foundation for their future training.

In 2004, to mark the fifth anniversary of the establishment of the institute for reporting medical errors in the United States, the five principles of patient safety were reviewed and amended, the most important of which was the amending of the second principle related to the method for systematically reporting of clinical errors [46]. This, in itself, indicates the importance of error reporting systems in reducing clinical errors. Shams al-Din has proposed that the establishing of effective systems for recording and reporting errors is a practical means to effectively reduce such errors [47]. In other studies, creation of a rapid response system for reporting clinical errors has been advocated [14].

According to our results, managerial problems such as lack of active participation of a hospital’s management officials in the controlling of errors and their self–centred perspective towards clinical errors have been considered as obstacles to the reporting of errors in the past and are categorized under organizational problems. While lack of active participation was not evaluated in prior investigations, emphasis has been put in previous studies on the self-centred perspective of managers [2, 32]. Furthermore, the hospital managers’ failure to demonstrate a suitable reaction with respect to clinical errors as has been indicated repeatedly throughout this study and in other previous studies. This has been associated with the inactive participation of high level managers in controlling clinical errors.

In our interviews with surgical assistants, clinical errors were regarded as a major problem in the healthcare system. Obstacles such as lack of emphasis on ethics, the inability to communicate effectively with patients, and lack of a precise definition of the probable types of errors that may occur during surgical residency training were grouped under “training problems” which were within “individual problems.”

Kuhpayehzadeh investigated the perspective of medical residents at Tehran University of Medical Sciences regarding the self-reporting of clinical errors and found that % 91 the medical residents were willing to learn how to identify and report clinical errors [48]. According to senior physicians, by creating a culture of error reporting, sharing personal experiences, and teaching professional ethics issues, training and self-reporting sessions can correct the attitude of medical assistants after graduation, making them more prone to reporting and correcting clinical errors. In this respect, using experienced consultants has been recommended in critical conditions [48].

Senior physicians’ reprimand is among the other codes specified at the level of the hospital. Previous studies show that % 68 of residents fear conflicts with senior physicians and do not therefore report clinical errors [48]. However, most senior physicians in university hospitals believe that the failure to report clinical errors is due to the lack of support by high-ranking officials in the education systems and the existing legal loopholes which are in contrast to the educational mission of training hospitals where surgical assistants should be taught what to do at the bedside of a patient. It is believed that policy-makers on the upper echelons of the health care system must alleviate the fear of reporting clinical errors in order to enhance the patient’s well-being and rectify the legal shortcomings and modify the existing laws accordingly.

### Limitations

One of the limitations of this study was that it was conducted in only one hospital. Thus, caution must be exercised while generalizing the results. Participants’ inadequate time for interviews was another limitation of this study.

## Conclusion

The results obtained from the present study show that in order to alleviate the obstacles hindering the reporting of clinical errors, individual and organizational problems should be resolved. It is proposed that in order to alleviate individual problems, the hospitals must train the nursing and medical staff on how to identify clinical errors, self-report them, and apply various methods to deal with and inform a patient upon the occurrence of a clinical error. In addition, it is recommended that the quality of reporting clinical errors be enhanced and the methods adopted for the systematic dealing with clinical errors be reinforced. In order to alleviate the problem of reporting clinical errors among physicians, a system for recording and following up clinical errors and addressing legal loopholes related to the reporting of clinical errors are also proposed.

## Data Availability

Data sharing is not applicable to this article as no datasets were generated or analysed during the current study.
